# Stable centromere positioning in diverse sequence contexts of complex and satellite centromeres of maize and wild relatives

**DOI:** 10.1186/s13059-017-1249-4

**Published:** 2017-06-21

**Authors:** Jonathan I. Gent, Na Wang, R. Kelly Dawe

**Affiliations:** 1 Department of Plant Biology, University of Georgia, Athens, USA; 2 Department of Genetics, University of Georgia, Athens, USA

**Keywords:** Centromere drift, Centromere stability, Satellite DNA, *CentC*, ChIP, CENP-A, cenH3

## Abstract

**Background:**

Paradoxically, centromeres are known both for their characteristic repeat sequences (satellite DNA) and for being epigenetically defined. Maize (*Zea mays mays*) is an attractive model for studying centromere positioning because many of its large (~2 Mb) centromeres are not dominated by satellite DNA. These centromeres, which we call complex centromeres, allow for both assembly into reference genomes and for mapping short reads from ChIP-seq with antibodies to centromeric histone H3 (cenH3).

**Results:**

We found frequent complex centromeres in maize and its wild relatives *Z. mays parviglumis*, *Z. mays mexicana*, and particularly *Z. mays huehuetenangensis*. Analysis of individual plants reveals minor variation in the positions of complex centromeres among siblings. However, such positional shifts are stochastic and not heritable, consistent with prior findings that centromere positioning is stable at the population level. Centromeres are also stable in multiple F1 hybrid contexts. Analysis of repeats in *Z. mays* and other species (*Zea diploperennis*, *Zea luxurians*, and *Tripsacum dactyloides*) reveals tenfold differences in abundance of the major satellite *CentC*, but similar high levels of sequence polymorphism in individual *CentC* copies. Deviation from the *CentC* consensus has little or no effect on binding of cenH3.

**Conclusions:**

These data indicate that complex centromeres are neither a peculiarity of cultivation nor inbreeding in *Z. mays*. While extensive arrays of *CentC* may be the norm for other *Zea* and *Tripsacum* species, these data also reveal that a wide diversity of DNA sequences and multiple types of genetic elements in and near centromeres support centromere function and constrain centromere positions.

**Electronic supplementary material:**

The online version of this article (doi:10.1186/s13059-017-1249-4) contains supplementary material, which is available to authorized users.

## Background

Eukaryotes segregate their chromosomes during cell division using spindle microtubules, where the microtubules attach to chromosomes via complex protein structures called kinetochores. Centromeres are the parts of the chromosomes where kinetochores assemble and are marked by specific DNA binding proteins, usually including the centromeric histone H3 variant cenH3 (also widely known as CENP-A) [1]. The size and sequence composition of centromeres, as defined by cenH3 footprints, varies widely between species. Centromeres have been reported from 40 kb in length in chicken [2] to 4 Mb in oat [3] and are usually dominated by tandem repeats (known as satellites) in plants and animals [4, 5]. In plants, a conserved family of *Gypsy* retrotransposons called centromeric retrotransposons specifically targets centromeres as well [6, 7]. In some species the chromosomes are holocentric (or polycentric) and characterized by multiple sites of centromere formation, and satellite DNA has been discovered in the polycentric centromeres of several plant genera [8, 9].

In most species the role of centromere sequence in conferring centromere function is unclear. With the exception of some fungi with small centromeres [10], no known centromere sequence motifs or structural features strictly define centromeres. Centromere sequences can vary widely even between homologous chromosomes in the same species [11, 12]. Some species, such as the African ass, potato, and maize, have a mixture of different types of centromeres, with some being rich in satellite DNA and others containing large numbers of retrotransposons and little or no satellite DNA [11, 13–16]. Centromeres without long arrays of satellite DNA have been referred to as evolutionarily new centromeres (ENCs) [15] and as neocentromeres [13]. These terms reflect the fact that centromeres have often been observed to form de novo at entirely new positions that lack satellite DNA [17]. Because of its innate instability, however, satellite DNA could also be lost from existing centromeres. Here we simply refer to centromeres that lack extensive satellite arrays as “complex” centromeres to describe the fact that they consist of a variety of retrotransposons and other polymorphic genetic elements. This sequence complexity, if assembled into a reference genome, allows for unambiguous mapping of short reads.

Experiments with human tissue culture and grass species have shown that the amount of cenH3/CENP-A loaded on centromeres is determined by cellular context rather than by the size or structure of the centromeric domains [18–20]. Consistent with this, overexpression of cenH3 (CID) in *Drosophila* causes ectopic centromere formation at multiple loci per chromosome [21]. However, the fact that cenH3 occupies smaller domains in neocentromeres than what is normally present on established centromeres suggests that sequence composition may also be important for centromere size and function [22–24]. Only about 100 CENP-A-containing nucleosomes occupy each centromere during cell division in human cells, leaving most of the nucleosomes to contain other forms of histone H3 [19]. During each cell cycle, the total amount of CENP-A is diluted by DNA replication but is replaced as a part of a self-propagating system of centromere maintenance where preexisting centromere proteins signal the deposition of new ones [1]. There are also mechanisms that remove cenH3. For instance, cenH3 removal occurs naturally as a part of plant gametogenesis [25, 26]. Similarly, budding yeast uses an E3 ligase-based mechanism to remove ectopically placed cenH3 from chromosome arms [27, 28]. It is likely that plants and animals have similar mechanisms to prevent the formation of ectopic centromeres and constrain normal centromere boundaries.

Large-scale genetic changes such as chromosomal rearrangements, deletions, and insertions or bursts of retrotransposon activity could force centromere positions to change. However, little is known about the dynamics and stability of centromeres at the purely epigenetic level. Centromeres in horse tissue culture cells were reported to occupy distinct positions ranging over a couple hundred kilobases, but the lines chosen may have also differed at the genetic level [29]. Perhaps the best evidence for epigenetic instability comes from chicken tissue cultured cells, where lines derived from a common parent showed clear evidence for centromere drifting on a scale of tens of kilobases [30]. The observed centromere movement occurred over an unknown number of cellular generations in wild-type chicken cells, but greater than 40. In contrast, lines containing mutants in key inner kinetochore proteins exhibited drift in as little as 40 cellular generations. We previously compared centromere positions between maize populations with genetically identical centromeres and found no evidence for centromere drift between populations, but left open the possibility of small scale drift between individual plants [31].

Here we take advantage of the complex centromeres in maize (*Zea mays mays*, the cultivated *Zea mays* subspecies) and a comparative analysis of its wild relatives to explore centromere dynamics and diversity in terms of both genetics (sequence composition) and epigenetics (cenH3 localization). Focusing on the B73 inbred stock, with its complex centromeres, we found that centromere boundaries are not rigidly defined, but ebb and flow between individuals, with visible differences on the order of hundreds of kilobases. These differences, however, were not shared between siblings, suggesting that positional shifts are generally not heritable. We found no evidence that centromere size or position were affected by centromeres at non-equivalent positions in inter- and intra-species F_1_ hybrids. We also found examples of complex centromeres in each of the three other *Z. mays* subspecies, with *Z. mays huehuetenangensis* having as many or more complex centromeres than *Z. mays mays.* In the genomes we sampled, the major satellite sequence *CentC* had a tenfold range in abundance, and sequences of individual *CentC* copies were highly polymorphic. The distributions of mutations in *CentC* copies were strikingly similar among species and subspecies. Surprisingly, polymorphic *CentC* copies were strongly enriched in CENH3 ChIP samples despite their dissimilarity to the consensus sequence. Taken together, these results indicate that centromeres can drift along the DNA as if untethered, but rather than progressing, maintain stable equilibrium over generations, suggesting a level of genetic control that is not apparent at the sequence level.

## Results

### Resilience of complex centromere positions to epigenetic drift and hybridization

The analysis of centromere positions and stability in maize relies primarily on the interpretation of ChIP-seq data aligned to the B73 reference genome. This is possible because most of the centromeres in B73 are complex, having little *CentC*, but large numbers of ancient retroelements that are nested within each other and effectively unique over the length of a 150-nucleotide Illumina read. However, three of the ten chromosomes in B73—1, 6, and 7—have large arrays of *CentC* that are presumably the locations of centromeres [14]. For these centromeres ChIP-seq fails for two reasons: because the physical map for these centromeres is incomplete and short reads cannot be uniquely mapped.

**Fig. 1:**
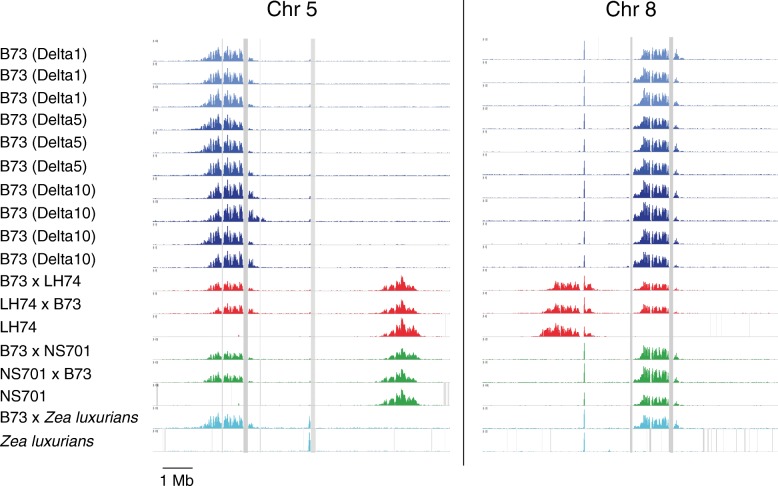
Analysis of CENH3 ChIP profiles in B73 lineages and F_1_ hybrids. ChIP-seq reads were mapped to the B73 genome, and the 10-Mb surrounding centromeres 5 (*left*) and 8 (*right*) are shown. *Vertical grey lines* indicate regions of zero coverage, including but not limited to gaps in the reference genome. Peaks are colored to indicate separate experiments; in cases where crosses were involved, the parent listed first was the female. Note that *Zea luxurians* has *CentC*-rich centromeres on chromosome 5 and 8, and as a result shows no unique alignment to the B73 reference (*CentC* was highly enriched in this ChIP experiment)

Previous ChIP-seq experiments in maize with antibodies to cenH3 (referred to as CENH3 in maize) demonstrated that different inbreds carrying genetically identical complex centromeres maintain the same centromere positions. The prior work assessed population averages derived from pools of 30–70 plants and left open the possibility that centromere positions might vary considerably among individuals [31]. To assay for such variation, we carried out ChIP on individual seedlings from three separate lineages of the B73 inbred stock. Each of the three lineages, called Delta1, Delta5, and Delta10, were derived from a single plant and maintained by 11 generations of self-crossing. Three or four siblings from each lineage were selected for ChIP. As expected, there were no overt differences in centromere positions among the three lineages (Fig. 1). There was, however, minor variation between siblings. The most extreme case was the second individual in the Delta10 lineage where the CENH3 read coverage on centromere 5 was higher on the right side of the centromere and lower on the left than its immediate siblings or any of the other individuals assayed. This positional shift was biological, not technical, as splitting the sample into two separate ChIPs produced identical read coverages (Additional file 1: Figure S1). These results demonstrate that CENH3 distributions are not rigidly fixed. Rather, variation exists but does not accumulate such that the average over a population remains stable.

We also wondered how centromeres would be affected by outcrossing. In *Arabidopsis thaliana*, cenH3 is erased from the egg cell such that only the sperm contributes cenH3 [25], raising the possibility that paternal centromeres specify the positions of both centromeres in the zygote. We previously identified two maize inbreds with centromeres at different positions relative to B73: NS701, where centromere 5 is at a different position; and LH74, where centromeres 5 and 8 are at different positions. We made bidirectional crosses between both inbreds and B73 and carried out ChIP-seq to test whether both parental centromere positions would be maintained in the hybrids or one position would shift to match the paternal one. We found that both positions were maintained with no evidence of any change in position (Fig. 1). We also carried out CENH3 ChIP-seq on an interspecies hybrid, B73 × *Zea luxurians. Z. luxurians* has a 50% larger genome and centromeres with long arrays of *CentC* [32, 33]. In the F_1_ hybrid, the centromeres on the B73 chromosomes neither shifted outside the normal range nor changed in size. Since these experiments were carried out specifically in F_1_ hybrids, it remains a possibility that centromere positions could shift or increase in size after subsequent generations in hybrid genetic backgrounds. These results, however, indicate that centromere positions are generally resilient to change when the genetic structure of the chromosome remains constant.

### Frequent complex centromeres in geographically and genetically diverse *Z. mays*

A recent study suggested that satellite-rich centromeres are the ancestral state in *Zea*, and that complex centromeres are an outcome of cultivation and inbreeding [13]. To look for complex centromeres in the wild, we carried out ChIP-seq on nine individual plants from three outcrossing *Zea* species (*mays*, *diploperennis*, and *luxurians*) and four *Zea mays* subspecies (*mays*, *parviglumis*, *mexicana*, and *huehuetenangensis*) (Table 1). Plants from at least two geographically diverse accessions were selected for each subspecies except *Z. mays huehuetenangensis*, which is native to a small region of western Guatemala. We also included one individual from the sister genus to *Zea*, *Tripsacum dactyloides*. The relationships between these species and subspecies have been well documented [34, 35]. *Z. mays parviglumis* is the most similar to *Z. mays mays* and is thought to be its closest relative. *Z. mays mexicana*, however, is known to hybridize with *Z. mays mays* in regions where both subspecies grow [36].

**Table 1:** Summary of CENH3 ChIP samples

Species/subspecies	Cultivar	Place of origin	Lineage	Plant ID	ChIP reads (in millions)	Input reads (in millions)	*CentC* in ChIP reads (%)	*CentC* in input reads (%)
*Zea mays mays*	B73	USA	Delta1(11.12)	Delta1-1	1.29	40.75	3.98	0.15
*Zea mays mays*	B73	USA	Delta1(11.12)	Delta1-2	11.55		2.15	
*Zea mays mays*	B73	USA	Delta1(11.12)	Delta1-3	6.19		5.05	
*Zea mays mays*	B73	USA	Delta5(11.12)	Delta5-3	1.62	31.45	2.82	0.12
*Zea mays mays*	B73	USA	Delta5(11.12)	Delta5-6	6.98		3.28	
*Zea mays mays*	B73	USA	Delta5(11.12)	Delta5-6	4.97		2.22	
*Zea mays mays*	B73	USA	Delta5(11.12)	Delta5-7	11.65		2.16	
*Zea mays mays*	B73	USA	Delta5(11.12)	Delta5-7	10.13		1.77	
*Zea mays mays*	B73	USA	Delta10(11.12)	Delta10-A	13.90		3.78	
*Zea mays mays*	B73	USA	Delta10(11.12)	Delta10-B	3.40	86.77	3.14	0.14
*Zea mays mays*	B73	USA	Delta10(11.12)	Delta10-8	5.59		3.99	
*Zea mays mays*	B73	USA	Delta10(11.12)	Delta10-D	4.35		1.93	
*Zea mays mays*	B73	USA	Delta10(11.12)	Delta10-D	5.70		2.97	
*Zea mays mays*	PI 628470	Oaxaca, Mexico		Oax-2	8.94	90.96	2.50	0.10
*Zea mays mays*	LH74	USA		LH74-1	10.67		3.74	
*Zea mays mays*	LH74 x B73	USA		LH74B73-3	8.95		3.03	
*Zea mays mays*	B73 x LH74	USA		B73LH74-1	7.13		3.62	
*Zea mays mays*	NS701	USA		J201-1	2.81		2.65	
*Zea mays mays*	B73 x NS701	USA		J202-4	2.93		2.78	
*Zea mays mays*	NS701 x B73	USA		J203-5	2.46		2.60	
*Zea mays mays* x *Zea luxurians*	B73 x PI 422162	USA		B73Lux	1.86	24.94	5.27	0.24
*Zea mays parviglumis*	Ames 21826	Guerrero, Mexico		26-1	5.25	43.65	7.54	0.28
*Zea mays parviglumis*	Ames 21889	Jalisco, Mexico		J205-3	2.90	36.91	5.52	0.30
*Zea mays mexicana*	Ames 8083	Federal District, Mexico		Mex-2	9.50	80.47	7.73	0.26
*Zea mays mexicana*	PI 566674	Durango, Mexico		74-1	5.44	49.19	5.08	0.30
*Zea mays mexicana*	PI 566677	Michoacan, Mexico		77-1	6.13	74.00	9.39	0.33
*Zea mays huehuetenangensis*	PI 441934	Heuhuetenango, Guatemala		Hue-2	9.07	87.92	0.83	0.05
*Zea mays huehuetenangensis*	PI 441934	Heuhuetenango, Guatemala		Hue-4	8.43	123.77	2.08	0.08
*Zea diploperennis*	PI 462368	Jalisco, Mexico		J179-1	1.82	33.57	13.53	0.33
*Zea luxurians*	PI 422162	Guatemala, via Florida		J178-3	1.92	56.45	11.05	0.37
*Tripsacum dactyloides*	PI 421612	USA		J180-3	2.18	81.02	14.61	0.48

**Fig. 2:**
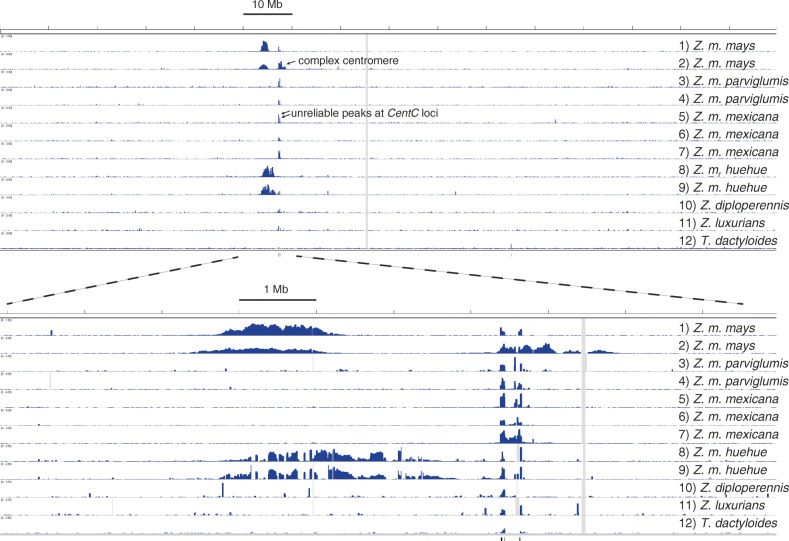
CENH3 ChIP enrichment on chromosome 9 in *Zea* and *Tripsacum*. ChIP and input reads were mapped to the B73 genome, and the ChIP enrichment relative to input is shown across chromosome 9 along with a zoomed in 10-Mb portion surrounding the centromere. *Tick marks* below the plots indicate positions of *CentC* in the genome assembly that can result in unreliable enrichment peaks. *Vertical grey lines* indicate regions of zero input coverage. Lines are as follows: 1) *Z. mays mays*, B73; 2) *Z. mays mays*, PI 628470; 3) *Z. mays parviglumis*, Ames 21889; 4) *Z. mays parviglumis*, Ames 21826; 5) *Z. mays mexicana*, Ames 8083; 6) *Z. mays mexicana*, PI 566674; 7) *Z. mays mexicana*, PI 566677; 8) *Z. mays huehuetenangensis*, PI 441934; 9) *Z. mays huehuetenangensis*, PI 441934; 10) *Z. diploperennis*, PI 462368; 11) *Z. luxurians*, PI 422162; 12) *T. dactyloides*, PI 421612. See Additional file 1: Figure S2 for all ten chromosomes

ChIP-seq data from *Zea mays* and its relatives were analyzed as 150-nucleotide, single-end Illumina reads aligned to the B73 reference genome. This approach makes it possible to identify conserved complex centromere regions, but cannot assess sequences that are present in the landrace or wild relative but absent in B73. Importantly for this analysis, we sequenced both ChIP and input reads and plotted enrichment (Fig. 2; Additional file 1: Figure S2). We have found that mapping ChIP reads alone can produce artificial peaks that appear to be complex centromeres (Additional file 1: Figure S3). In addition, the number and complexity of tandem repeats are poorly represented in the genome reference sequence. Thus, they produce artificially high and unreliable read coverage at loci where they are included in the genome reference sequence even for reads that are mapped uniquely. The results, however, clearly revealed examples of complex centromeres in all four *Z. mays* subspecies, including an outcrossing *Z. mays mays* accession from Oaxaca, Mexico (PI 628470). The data from *Z. mays huehuetenangensis* were the most striking, showing complex centromeres on every chromosome. However, for most chromosomes in subspecies other than *huehuetenangensis*, the ChIP-seq data showed very small or no centromeric enrichment patterns when aligned to B73 chromosomes, suggesting that the centromeres were composed almost entirely of *CentC*. We also mapped the ChIP-seq data to a genome assembly of the W22 inbred and found that the number of visible complex centromeres was identical for B73 and W22 (data not shown), though we do not expect all complex centromeres, particularly in the non-*mays* species, to be detectable without their own reference genome sequences.

**Fig. 3:**
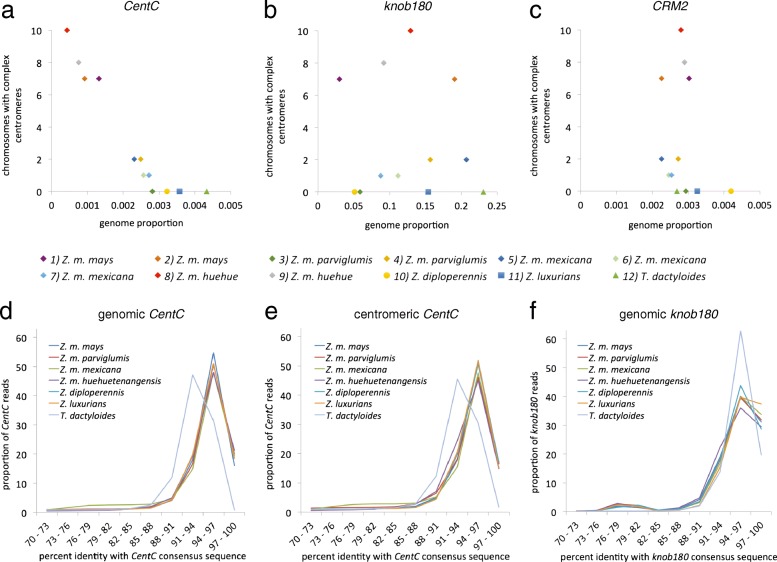
Analysis of genomic and centromere-associated *CentC*. **a**–**c** Number of complex centromeres as a function of abundance of *CentC*, *knob180*, and *CRM2* elements in the genome. Complex centromeres are indicated by peaks of CENH3 enrichment of >500 Kb in length in Fig. 2. Two peaks on a chromosome (potentially explained by heterozygosity) count for only one complex centromere. Full sample names are as follows: 1) *Z. mays mays*, B73; 2) *Z. mays mays*, PI 628470; 3). *Z. mays parviglumis*, Ames 21889; 4) *Z. mays parviglumis*, Ames 21826; 5) *Z. mays mexicana*, Ames 8083; 6) *Z. mays mexicana*, PI 566674; 7) *Z. mays mexicana*, PI 566677; 8) *Z. mays huehuetenangensis*, PI 441934; 9) *Z. mays huehuetenangensis*, PI 441934; 10) *Z. diploperennis* PI 462368; 11) *Z. luxurians* PI 422162; 12) *T. dactyloides*, PI 421612. **d** Identity with *Zea* consensus sequence in genomic *CentC* copies, as sampled in input reads. **e** Identity with *Zea* consensus sequence in centromeric *CentC* copies, as sampled in ChIP reads. **f** Identity with *Zea* consensus sequence in genomic *knob180* copies, as sampled in input reads

The amount of *CentC* in the sampled genomes (revealed by analysis of the input reads) varied tenfold, from a proportion of 0.043% in Z. mays *huehuetenangens*is to 0.433% in *Tripsacum* (Fig. 3a). We speculated that centromeres would be preferentially located at large arrays of *CentC* when they are available. In support of this, the abundance of *CentC* in the sampled genomes negatively correlated with numbers of detectable complex centromeres (Fig. 3a). The abundance of centromeric retrotransposons (*CRMs*) did not correlate with existence of complex centromeres, nor did abundance of a non-centromeric satellite, *knob180* (Fig. 3b, c; Additional file 1: Figure S4).

### *CentC* polymorphism and relation to CENH3 binding

Alpha satellites in human cells have been reported to show evidence of homogenization, presumably as a result of repeated expansion and contraction of long identical repeat arrays by unequal recombination [37]. This differs from maize, where, at least in the B73 inbred, *CentC* repeats are highly polymorphic and show no signs of homogenization nor accumulation of specific variants [33]. We wondered whether genomes with large amounts of *CentC* (Fig. 3a) would show evidence of *CentC* homogenization similar to alpha satellites in humans. To this end we took advantage of RepeatExplorer software, which identifies repeats without using a reference that may bias the results [38]. Surprisingly, all *Zea* species and subspecies examined had the same consensus *CentC* sequence and a similar level of *CentC* polymorphism, where the overall level of identity with the consensus was close to 96% (Fig. 3d). Very few *CentC* copies were identical to the consensus throughout the *Zea* samples, providing no evidence for sequence homogenization within *CentC* arrays. In contrast, copies of the tandem repeat *knob180* found on maize chromosome arms, which is under selection for meiotic drive in some backgrounds [39], showed higher levels of identity to its consensus sequence (Fig. 3f).

The observation that *CentC* is generally conserved (though polymorphic) and reliably present in the centromeres of *Zea* and *Tripsacum* raises the question of whether it makes a useful contribution to centromere function. One way it might contribute is by making a good substrate for CENH3 binding (though CENH3 is clearly not limited to *CentC*). If this were the case, we would expect to see a different distribution of *CentC* variants from ChIP than whole-genome input. The simplest expectation would be that the fraction of *CentC* copies bound to CENH3 would more closely resemble the consensus. However, plots of CentC polymorphism in ChIP and input samples produced nearly identical patterns (Fig. 3d, e). Consistent with this, k-mer analysis of *CentC* sequences in the reads revealed similar frequencies of distinct k-mers both between species and between ChIP and input reads (Additional file 1: Figure S5). For this analysis, we sampled the same number of *CentC* reads from each species (30,000), trimmed all reads to the same length (100 nucleotides), and counted the frequency (copy number) of distinct 50-mers. With this sampling depth, *CentC* reads derived from a genome with perfectly homogenous *CentC* would yield distinct 50-mers with an average copy number of 9808 each (51 50-mers per read times 30,000 reads divided by 156 possible 50-mers in a 156-bp circular sequence). In contrast, in each species that we examined, the vast majority of 50-mers were in copy numbers of less than 100, with a tail of the distributions reaching up to copy numbers of 2000. Taken together the results indicate that although *CentC* is the predominant genetic substrate for centromeres, any functional contribution has a very loose relation to its linear sequence.

## Discussion

CENH3 is diluted during DNA replication and replenished later in the cell cycle by a mechanism that relies on the presence of other kinetochore proteins [40, 41]. During the growth of a maize plant from a single-cell zygote to the next generation, this dilution/replenishment process occurs around 50 times [42]. In the absence of sequence-specific binding of CENH3 to DNA, one would expect that changes in the distribution of CENH3 nucleosomes would accumulate between individual cells. While we cannot measure CENH3 distributions in individual cells, we can measure the average CENH3 distribution in large numbers of cells derived from two cells (whole seedlings derived from a single egg and sperm). The variation in centromere positions we observe between seedlings (Fig. 1) could be largely determined by the initial position in the zygote or could accumulate throughout development. Consistent with our prior work, we found no evidence of heritable variation between genetically identical individuals, which confirms our conclusion that the genetic makeup of the centromere constrains the average centromere position [31]. This constraint is not easily loosened, neither by parent-of-origin affects in *Z. mays mays* nor by hybridization, including interspecies hybridization between *Z. mays* and *Z. luxurians.*

Satellite centromeres were more common than complex centromeres in our survey of *Zea*, except in *Z. mays mays* and *Z. may huehuetenangensis* (Fig. 2). This is consistent with FISH karyotyping of diverse *Zea* that indicates less *CentC* in these subspecies than in other *Zea* [14]. An inverse relationship between the amount of *CentC* on a chromosome and the presence of a complex centromere has been clearly demonstrated in *Z. mays mays*, the cultivated subspecies [13]. This could be explained if the loss of *CentC* induces the formation of the complex centromeres [13] or if the formation of complex centromeres induces the loss of *CentC* by exposing them to recombination. We cannot rule out either hypothesis, but our data show that complex centromeres are more common throughout *Zea mays* when there is less *CentC*. While it makes sense that centromeres must occupy more complex regions if there are no tandem repeat arrays, the reverse need not be true. For instance, in the B73 inbred, centromere 5 does not overlap with the only mapped *CentC* array on chromosome 5 [33] and large arrays of *CentC* are visible by FISH in other maize chromosome arms [3, 43]. Our observation that, as a general rule, centromeres do tend to occupy long *CentC* arrays when they are present (Fig. 3) supports the hypothesis that *CentC* is particularly well adapted for centromere function [44, 45]. No empirical studies have addressed the role of tandem repeats in plant centromeres, but several studies of animal cells have demonstrated subtle defects in neocentromeres that lack normal centromeric tandem repeats [24, 46–48].

Our data raise many interesting questions about the dynamics of *CentC*. How is the *CentC* consensus sequence conserved across *Zea* when individual copies are highly polymorphic and the number of copies varies dramatically between subspecies, between individuals, and even between homologous chromosomes (Fig. 3)? Why is *CentC* conserved in distant grasses such as *Oryza* yet absent from closer ones such as *Sorghum* and *Miscanthus* [5]? Do *Z. mays mays* and *Z. mays huehuetenangensis* have more complex centromeres because of recent loss of *CentC* or are they better representations of an ancestral type that had little or none (and which would have been like *Sorghum* and *Miscanthus*)? Are these repeats frequently transferred horizontally, as centromeric retrotransposons are proposed to do [6]? Perhaps the most important question is whether *CentC* contributes towards centromere function, or at the other extreme, whether it is merely a selfish element that hijacks centromeres. Our comparison of *CentC* copies in the centromere (ChIP) versus *CentC* copies in the whole genome (input) revealed no overt preference for the conserved *CentC* consensus sequence in centromeres. Thus, any contribution of *CentC* to centromere function must allow for a good deal of flexibility in *CentC* sequence. These data along with our experiments showing stable positioning of complex centromeres demonstrate a general principle of centromeres, which is that they can be stably propagated over a wide diversity of centromere genetic elements.

## Conclusions

**Fig. 4:**
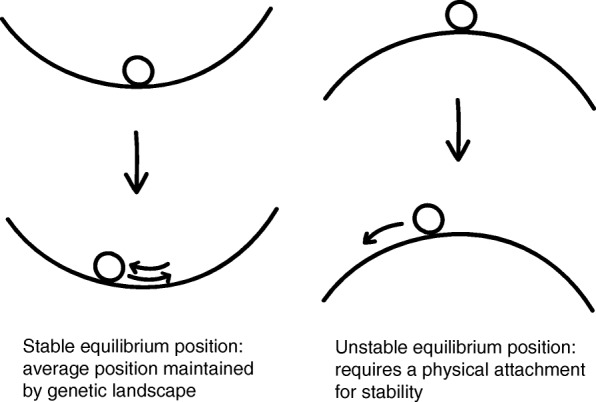
Grape in a bowl analogy for centromere positioning. In this model, centromere positioning is determined by equilibrium points

To explain how centromere positions are not defined by DNA sequence yet are tightly constrained, it may be helpful to think about centromeres in terms of the grape-in-a-bowl analogy (Fig. 4). Even if the grape is bumped or the bowl is rocked, the grape will maintain its position, on average. This does not require a physical attachment between the grape and the bowl because the grape’s location is a point of stable equilibrium. Similarly, centromere positioning does not require sequence-specific binding of proteins to DNA, but is instead a product of landscapes of genetic elements on the chromosome that creates points of stable equilibrium. An alternative case, of an unstable equilibrium, would require a physical attachment to avoid the grape changing its position. This might be analogous to the centromeres of budding yeasts, where the position of the single cenH3-containing nucleosome in each centromere is specified by DNA sequence [49].

What are the features of the genetic landscape that determine points of stable equilibrium? Several phenomena have been proposed. One is a negative role of transcribed genes. Centromeres are usually located in gene-poor regions, and evidence from experiments with maize chromosomes suggests genes can help enforce centromere boundaries [3]. The existence of megabase-scale arrays of non-centromeric tandem repeats such as the *knob180* repeats in maize indicates that absence of genes is not sufficient to promote centromere formation [50]. A second candidate feature is a positive role for transcription. At first glance this seems like a contradiction; however, a specific form of transcription could occur in tandem repeats that, for example, facilitates incorporation of CENH3 [1]. The sequence features that are important for such transcription might be highly flexible and thus would not be expected to be conserved. A third feature could be related to DNA repair by homologous recombination [51]. Multiple centromere proteins are related to homologous recombination and DNA repair [52]. The tandem repeats of human centromeres (alpha satellites) exhibit a specialized form of DNA repair, including the formation of DNA loops that might be important for centromere organization [53]. Last is the potential for tandem repeats to form strong interactions between DNA and cenH3 nucleosomes, which might be important to tolerate the stresses associated with spindle attachments. This theory has been discussed but not tested [44, 45, 54]. It is likely that multiple features—scarcity of genes, a specific form of non-coding transcription, DNA repair by homologous recombination, and strong nucleosome-DNA interactions—work together to subtly influence the position and function of centromeres in a way that tolerates multiple sequence contexts.

## Methods

### Plant materials

All accessions with PI or Ames numbers were obtained from GRIN, the National Genetics Resource Program (Ames, Iowa). Accession names and geographical origins are indicated in Table 1.

### ChIP and library preparation

Whole seedlings including roots between 3 and 13 g in weight were harvested and frozen in liquid nitrogen, then finely ground with pre-chilled mortars and pestles. Between 3 and 4 g of each were used for ChIP using a native ChIP protocol with micrococcal nuclease digestion of the DNA. An antibody raised against rice CENH3, which has broad reactivity to CENH3 in grasses, including oat, wheat, millet, and maize, was used to immunoprecipitate single nucleosomes containing CENH3 [3, 18, 55]. A detailed, step-by-step protocol is included in Additional file 2. For each ChIP, 5–30 ng of DNA was used for preparing Illumina sequencing libraries (KAPA hyper prep kit #KK8500). Barcoded adapters were used for pooling libraries (Bioo Scientific NEXTflex™ Bisulfite-Seq Barcodes, #511912). Libraries were amplified with five or six cycles of PCR, and amplicons of 100–200 bp were separated from longer fragments by gel electrophoresis and purified without heating (Qiagen QIAquick Gel Extraction Kit #28704). The Illumina NextSeq500 platform was used to generate 150-nucleotide single-end reads, and numbers of reads for each sample are listed in Table 1.

### Data analysis

Reads were quality trimmed using the FASTX-Toolkit 0.0.14 fastq_quality_trimmer, with “-Q33 -t 20” parameters (http://hannonlab.cshl.edu/fastx_toolkit/), then adapters removed with Cutadapt with the following parameters: “-a AGATCGGAAGAGC -m 100 -e .05 -O 1 -m 100” [56]. Reads were mapped to the B73 refgen V4 genome [57] and the W22 version 2.0 assembly (http://www.maizegdb.org/genome/genome_assembly/Zm-W22-REFERENCE-NRGENE-2.0) using the Burrows-Wheeler Aligner BWA-MEM with default parameters [58]. Only uniquely mapping reads, defined by MAPQ values of at least 20, were included for further analysis. The alignments were converted to BAM files and sorted using SAMtools [59]. Read coverage and enrichment were displayed after converting BAM files to tdf files with means of 20,000 kb intervals using the Integrative Genome Viewer [60]. RepeatExplorer [38] was used to cluster reads independently of genome alignment from each input sample (default parameters). Circular consensus sequences from each set of reads in the *CentC* cluster produced by RepeatExplorer were made using the Geneious® version 8.0.4 De Novo Assemble tool with default “High Sensitivity/Medium” settings (with the following options selected: “Don't merge variants with coverage over approximately 6”, “Merge homopolymer variants”, and “Circularize contigs with matching ends”). The abundance and percent identity with consensus sequences of repeats in the ChIP and input files was determined using blastall with parameters as follows: “-p blastn -e 1e-5 -W 7 -G 2 -E 1 -r 1 -q -1”. Only reads producing alignments of at least 125 bp in length to consensus sequence dimers were included. The *Zea CentC* consensus sequence shared in all *Zea* genomes sampled and used for these analyses is:

TGGTTCCGGTGGCAAAAACTCGTGCTTTGTATGCACCCCGACACCCGTTTTCGGAATGGGTGACGTGCGGCAACGAAATTGCGCGAAACCACCCCAAACATGAGTTTTGGACCTAAAGTAGTGGATTGGGCATGTTCGTTGCGAAAAACGAAGAAA.

The corresponding *knob180* consensus sequence is:

TGGGGTGAGGTGTATGAGCCTCTGGTCGATGATCAATGGCCACACAACCCCCATTTTTGTCGAAAATAGCCATGAACGACCATTTTCAATAATACCGAAGGCTAACACCTACGGATTTTTGACCAAGAAATGGTCTCCACCAGAAATCCAAGAATGTGATCTATGGCAAGGAAACATATG.

JELLYFISH software, version 2.2.3, was used for k-mer analysis [61]. After adapter removal, reads were trimmed to 100 nucleotides and aligned to the *CentC* consensus dimer sequence as before, except only reads producing alignment lengths of at least 90 bp were included for subsequent analysis. We sampled 30,000 *CentC* reads from each species ChIP and input.

## Additional files

Additional file 1: Figure S1. Apparent variation in CENH3 distributions is biological, not technical. **Figure S2.** CENH3 ChIP enrichments on 100-Mb regions of each chromosome. **Figure S3.** ChIP-seq read coverage verses enrichment. **Figure S4.** Abundance of centromeric retrotransposons and relation to frequency of complex centromeres. **Figure S5.** *CentC* k-mer analysis. (PDF 5184 kb)

Additional file 2: ChIP protocol. (DOCX 132 kb)

Additional file 3: Sequence Read Archive run IDs. (XLSX 41 kb)
