# Supplementary material for: Stable centromere positioning in diverse sequence contexts of complex and satellite centromeres of maize and wild relatives
Source: Genome Biol. 2017 Jun 21;18:121. doi: 10.1186/s13059-017-1249-4 (PMC5480163; doi:10.1186/s13059-017-1249-4)
Supplement: Supplementary file 1 — Apparent variation in CENH3 distributions is biological, not technical. Figure S2. CENH3 ChIP enrichments on 100-Mb regions of each chromosome. Figure S3. ChIP-seq read coverage verses enrichment. Figure S4. Abundance of centromeric retrotransposons and relation to frequency of complex centromeres. Figure S5. CentC k-mer analysis. (PDF 5184 kb) [file 13059_2017_1249_MOESM1_ESM.pdf]

Figure S1

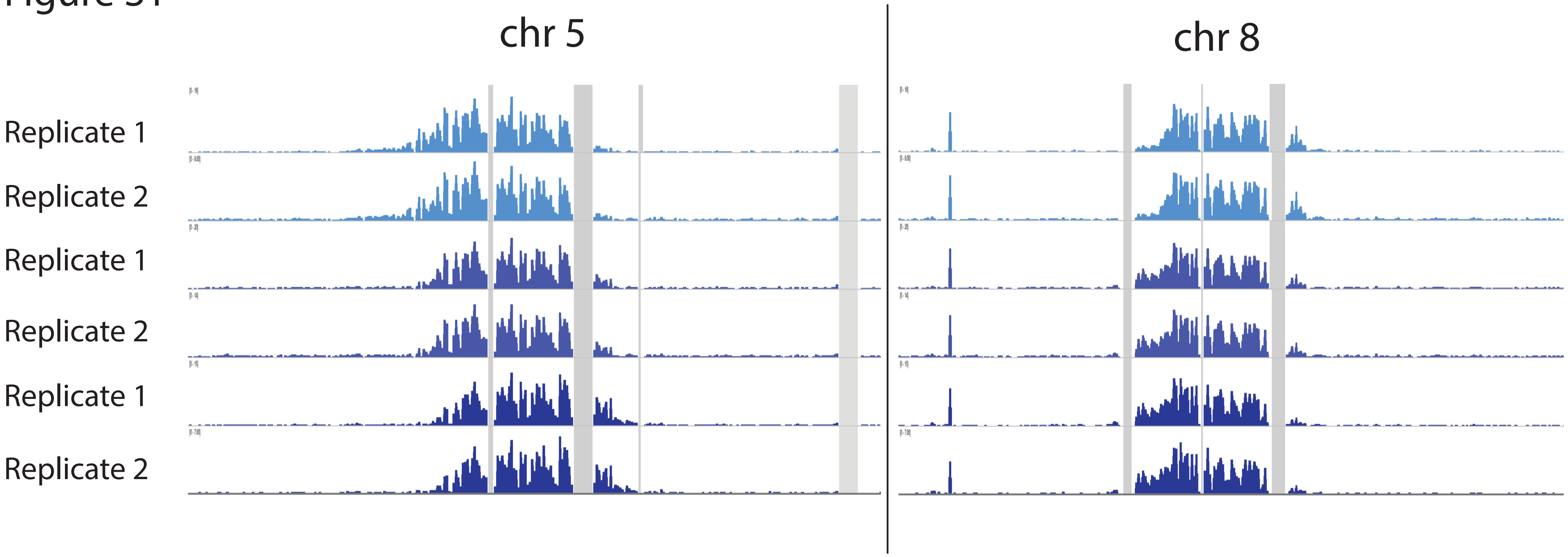

Figure S1. Apparent variation in CENH3 distributions is biological, not technical.

CENH3 ChIP reads were mapped to the B73 genome and coverage was displayed on 10 Mb regions of chromosomes 5 and 8. One Delta1 and two Delta2 plants from Figure 1 were split into two ChIPs each. Vertical grey lines indicate regions of zero coverage, including but not limited to gaps in the reference genome.

Figure S2

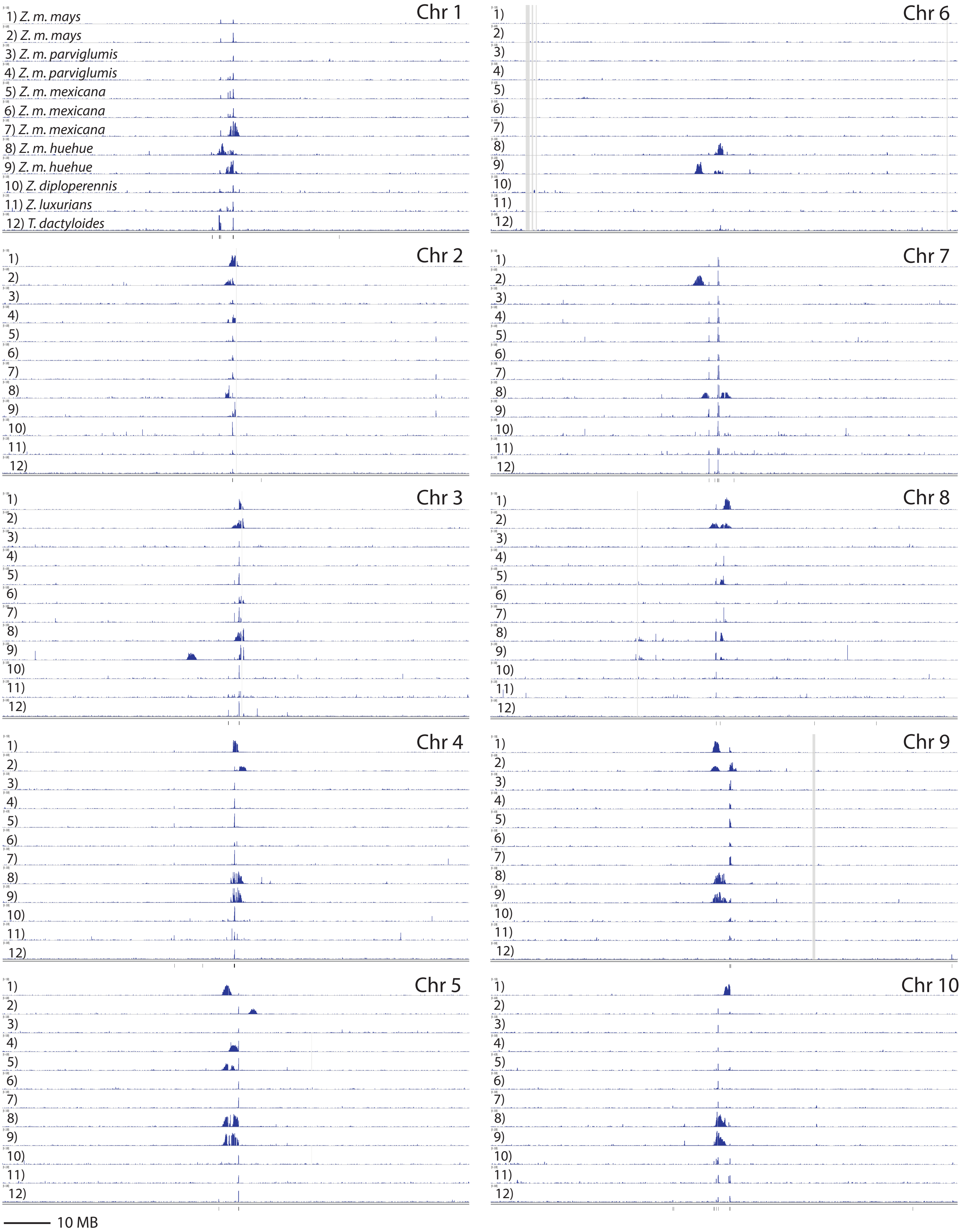

Figure S2: CENH3 ChIP enrichments on 100 Mb regions of each chromosome. ChIP and input reads were mapped to the B73 genome and the ChIP enrichment shown for 100 Mb regions surrounding each centromere. Tick marks below the plots indicate positions of *CentC* in the genome assembly that cause artifactual enrichment peaks. Lines are as follows: 1) *Z. mays mays*, B73. 2) *Z. mays mays*, PI 628470. 3) *Z. mays parviglumis*, Ames 21889. 4) *Z. mays parviglumis*, Ames 21826. 5) *Z. mays mexicana*, Ames 8083. 6) *Z. mays mexicana*, PI 566674. 7) *Z. mays mexicana*, PI 566677. 8) *Z. mays huehuetenangensis*, PI 441934. 9) *Z. mays huehuetenangensis*, PI 441934. 10) *Z. diploperennis,* PI 462368. 11) *Z. luxurians,* PI 422162. 12) *T. dactyloides*, PI 421612. Vertical grey lines indicate regions of zero input coverage.

Figure S3

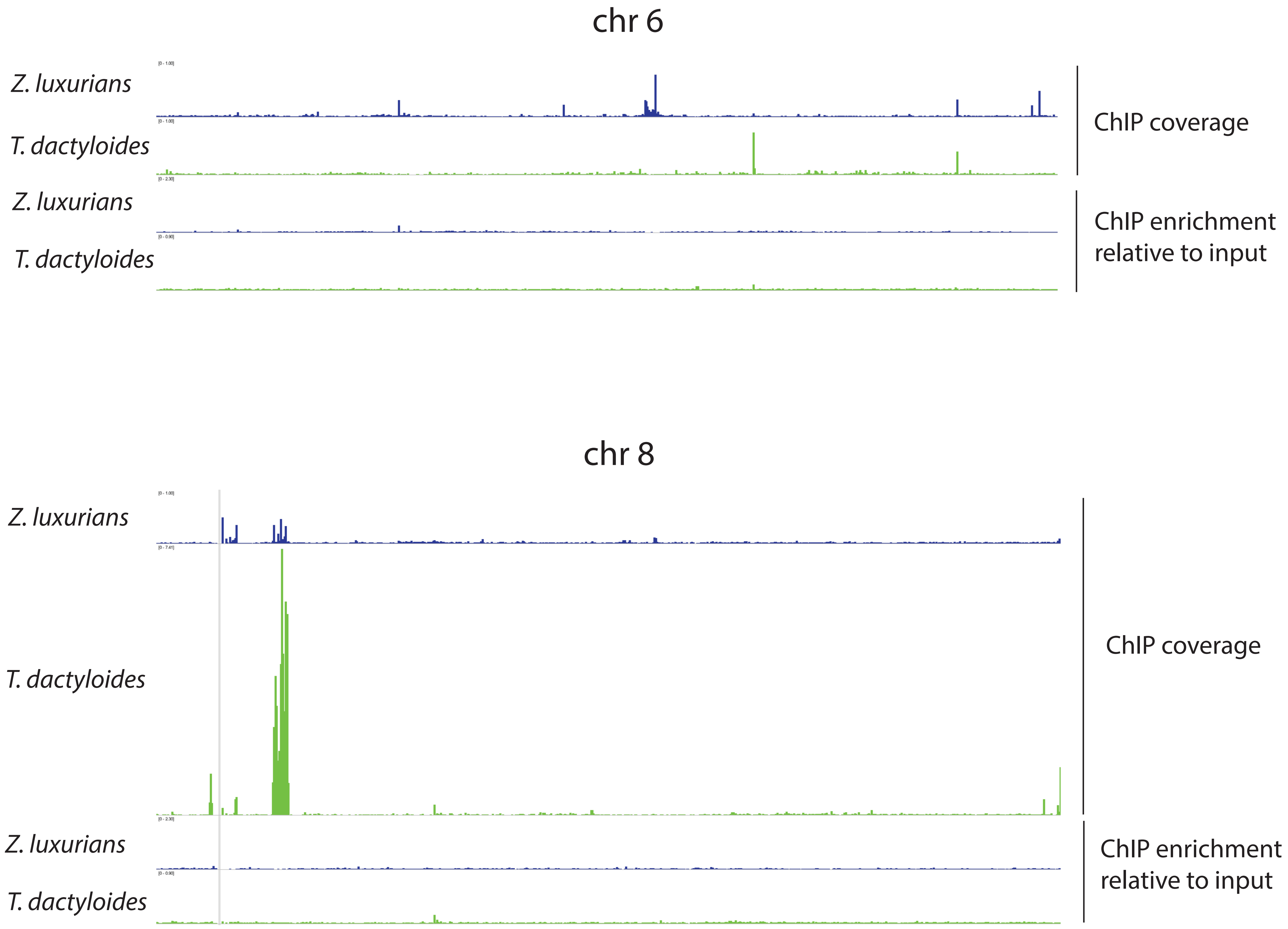

Figure S3. ChIP-seq read coverage verses enrichment

Coverage and enrichment are displayed separately on 100 Mb regions of chromosomes 6 and 8. Coverage is the distribution of ChIP-seq reads aligned to the physical map without regard to the input. Enrichment is the ratio of ChIP coverage to input coverage.

Figure S4

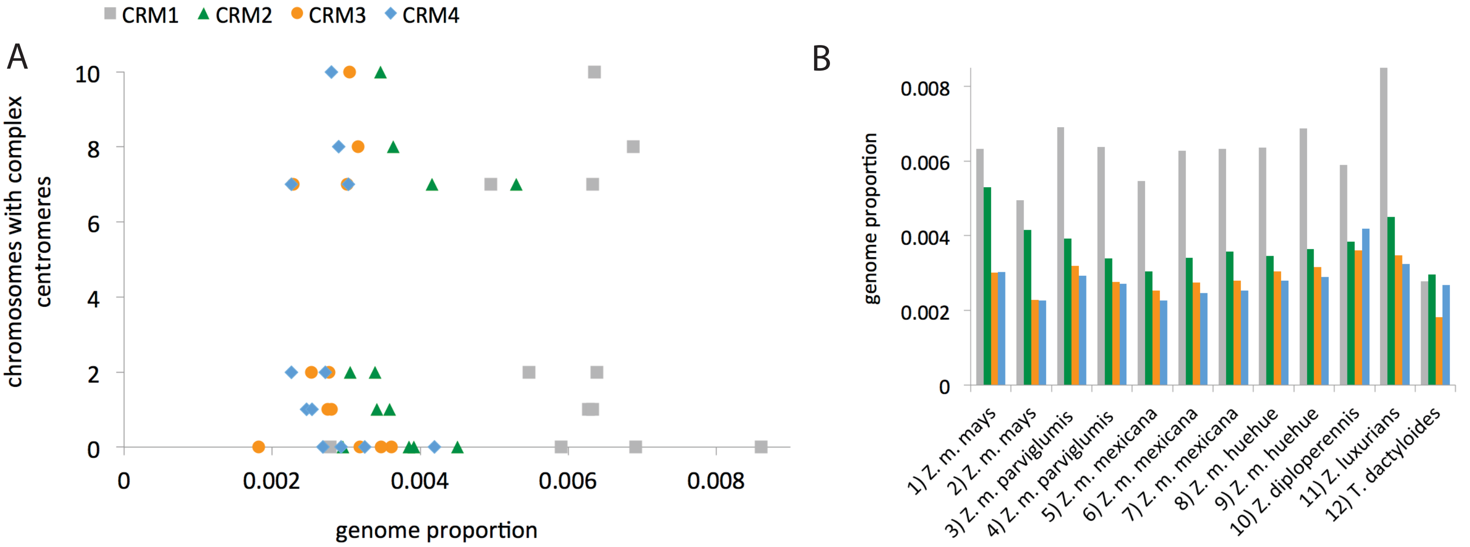

Figure S4. Abundance of centromeric retrotransposons and relation to frequency of complex centromeres

(A) Input reads from each sampled genome were blasted to reference centromeric retrotransposons sequences (*CRM1-4*) to estimate their abundances and then plotted relative to frequency of visible complex centromeres, as determined by mapping ChIP reads to the B73 genome (see Figure 2).
(B) *CRM* abundances from (A) are shown for each genome separately: 1) *Z. mays mays*, B73. 2) *Z. mays mays*, PI 628470. 3) *Z. mays parviglumis*, Ames 21889. 4) *Z. mays parviglumis*, Ames 21826. 5) *Z. mays mexicana*, Ames 8083. 6) *Z. mays mexicana*, PI 566674. 7) *Z. mays mexicana*, PI 566677. 8) *Z. mays huehuetenangensis*, PI 441934. 9) *Z. mays huehuetenangensis*, PI 441934. 10) *Z. diploperennis,* PI 462368. 11) *Z. luxurians,* PI 422162. 12) *T. dactyloides*, PI 421612. Colors are the same as in (A).

Figure S5

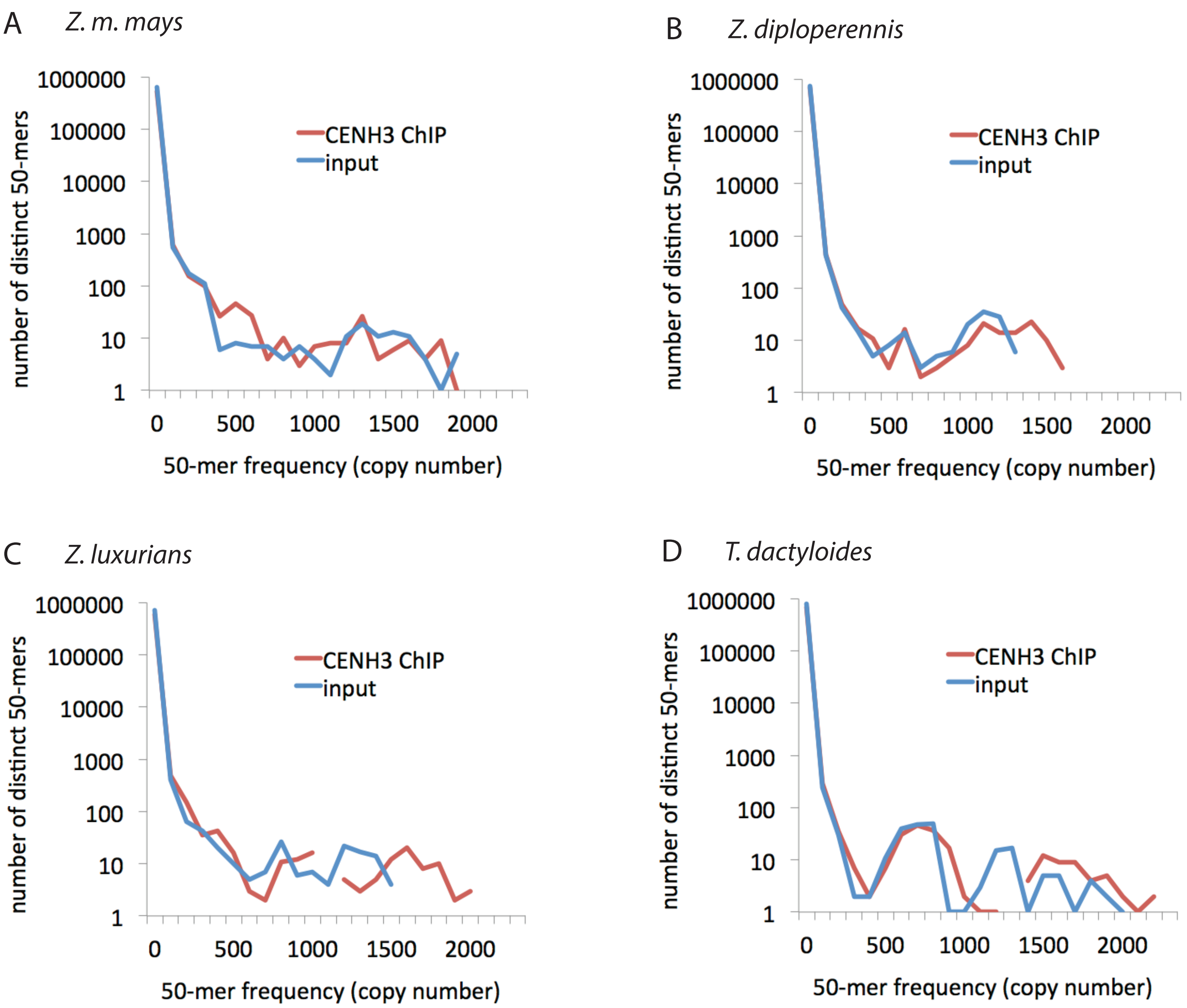

Figure S5: *CentC* k-mer analysis

K-mer frequency distributions of *CentC* reads in **(A)** *Z. mays mays*, B73, **(B)** *Z. diploperennis*, PI 462368, **(C)** *Z. luxurians*, PI 422162, and **(D)** *T. dactyloides*, PI 421612. Due to the logarithmic Y-axis, values of zero produce discontinuties in the plots. All reads were trimmed to 100-nt and aligned by BLAST to the *Zea CentC* consensus to identify *CentC* reads. Only reads that produced alignment lengths of at least 90 bp were included in the analyis. 30,000 *CentC* reads were sampled from each species, and the number of distinct k-mers was counted using JELLYFISH software.
