# Supplementary material for: Stable centromere positioning in diverse sequence contexts of complex and satellite centromeres of maize and wild relatives
Source: Genome Biol. 2017 Jun 21;18:121. doi: 10.1186/s13059-017-1249-4 (PMC5480163; doi:10.1186/s13059-017-1249-4)
Supplement: Supplementary file 2 — ChIP protocol. (DOCX 132 kb) [file 13059_2017_1249_MOESM2_ESM.docx]

Dawe Lab 2016 CENH3 Native Chromatin Immunoprecipitation (ChIP) Protocol

**Day 1. Preparation (1 hr)**

**1.** Grind 3-5 grams frozen maize tissue to a fine powder using liquid nitrogen. Store at -80°C in 50ml conical tubes. (Note: thorough grinding of the tissue is key to efficient nuclear isolation).

**2.** Make M1, M2, and Elution buffers fresh on day of experiment. Other buffers can be made months in advance. Put M2 buffer, MNB, incubation buffer and isopropanol in a 4°C fridge and 70% ethanol in a -20°C freezer.

**3.** Chill a swinging bucket centrifuge to 4°C.

**Chromatin extraction and MNase digestion (2 hr)**

**4.** Add 20 ml of M1 Buffer (room temperature) to ground tissue (-80°C) and thoroughly mix.

**5.** Rock the tube at 4°C for 10 min.

**6.** In a 4°C room, filter ground tissue solution through two layers of Miracloth and transfer to a new 50ml conical tube. Gently squeeze fluid through Miracloth.

**7.** Centrifuge at 1,200 × g for 10 min at 4°C using swinging bucket rotor and pour off supernatant.

**8.** Add 10 ml of M2 Buffer (4°C) and gently suspend pellet by pipetting through a tip with a widened opening (cut it back with scissors) while on ice.

**9.** Centrifuge at 1,000 × g for 10 min at 4°C using swinging bucket rotor and pour off supernatant.

**10.** Repeat previous two steps twice for a total of three M2 Buffer washes. On the third wash, use 5 rather than 10 ml of M2 Buffer.

**11.** Add 5 ml MNB (4°C) and gently suspend pellet by pipetting through a cut tip while on ice.

**12.** Centrifuge at 1,000 × g for 10 min at 4°C using swinging bucket rotor and pour off supernatant. Blot tube on paper towel to remove more supernatant.

**13.** Add 650 μl MNB (4°C) and gently suspend pellet by pipetting through a cut tip while on ice.

**14.** Transfer nuclei in MNB to a 1.5ml microcentrifuge tube and centrifuge at 1,000 × g for 2 minutes at 4°C.

**15.** Estimate volume of pellet and add 1 μl of MNase (micrococcal nuclease; 2,000,000 gel units/ml) per 50 μl of pellet and mix thoroughly by pipetting while on ice. (Note: good nuclei extractions will produce >100 μl of nuclei, more than enough for two ChIPs. An extra 650 μl MNB can be added here and the sample slit in two, or later on before adding Incubation Buffer).

**16.** Incubate 4 min in 37°C water bath. Invert several times 2 minutes in.

**17.** Immediately place on ice, add 6.5 μl 0.5 *M* EDTA, and mix.

**Input DNA purification and start of overnight antibody incubation (2 hr)**

**18.** Centrifuge digested chromatin at >=16,000 × g for 10 min at 4°C.

**19.** During centrifugation, add protease inhibitors to Incubation Buffer: 2 μl of 0.1 *M* PMSF and 10 μl of proteinase inhibitor cocktail per ml.

**20.** Combine 650 μl of MNase-digested supernatant from step 18 with 650 μl Incubation Buffer in a new 1.5ml or 2ml microcentrifuge tube.

**21.** Purify 13 μl of the remaining MNase-digested supernatant from step 18 (input) by combining it with 800 μl Elution Buffer and 800 μl phenol/chloroform/isoamyl alcohol 25:24:1 in a 2ml microcentrifuge tube, vortexing for 30 sec, and centrifuging at >=16,000 × g for 4 min.

**22.** Transfer supernatant to a 1.5ml microcentrifuge tube and precipitate input DNA with 80 μl of 3 *M* sodium acetate, 2 μl of 20 mg/ml glycogen, and 480 μl of isopropanol (4°C). Mix thoroughly and leave at room temperature for 10 min.

**23.** During precipitation, cast a 2% agarose gel with a fine-tooth comb for wells.

**24.** Centrifuge at >=16,000 × g for 10 min at 4°C.

**25.** Remove supernatant, add 1 ml cold 70% ethanol, and dislodge pellet by flicking and inverting tubes.

**26.** Centrifuge at >=16,000 × g for 1 min at 4°C.

**27.** Remove supernatant, quick spin, and pipet out all residual supernatant.

**28.** Air dry at room temperature for 3-5 minutes or until pellet just becomes clear, then add 20 μl of 10 *mM* Tris-HCl, pH 8.0.

**29.** Run 2 μl of purified input DNA on a 2% agarose gel just far enough to resolve 100-bp marker bands in the 100-500 bp range. A good MNase digestion will produce a series of visible bands starting at ~160 bp and increasing in size by ~190 bp increments. Stronger digestions that result in mainly a single 160 bp or smaller band will bias the results toward GC rich DNA [Gent et al 2014].

**30.** Add 5 μl of 2.1 mg/ml antibody to the 1300 μl volume of MNase-digested chromatin and rotate overnight at 4°C.

**Day 2. Isolation of antibody-bound protein-DNA complex with rProtein A sepharose (3.5 hr)**

**31.** Using a cut pipet tip, put 300 μl of rProtein A sepharose in a 2ml microcentrifuge tube.

**32.** Add 900 μl of Incubation Buffer (4°C, no proteases added) and mix.

**33.** Centrifuge at 2,400 × g for 1 min and remove supernatant.

**34.** Repeat previous two steps.

**35.** Transfer the chromatin-antibody solution that incubated overnight at 4°C to the tube containing the washed rProtein A sepharose and rotate for 3 hr at 4°C.

**36.** Warm Elution Buffer to at least 42°C for later use.

**37.** Centrifuge chromatin-antibody-sepharose solution at 2,400 × g for 1 min, carefully remove all supernatant, and place on ice. (Note: supernatant contains DNA that is not attached to the antibody complex and will reduce ChIP enrichment if not carefully removed, here and in the following four washes).

**38**. Add 1 ml of Wash Buffer A (4°C), mix, centrifuge at 2,400 × g for 1 min, carefully remove all supernatant, and place on ice.

**39**. Wash again with 1 ml of Wash Buffer A, as in previous step.

**40**. Add 1 ml Wash Buffer B (4°C), mix, centrifuge at 2,400 × g for 1 min, carefully remove all supernatant, and place on ice.

**41**. Wash again with 1 ml of Wash Buffer B, as in previous step.

**ChIP DNA purification (1.5 hr)**

**42**. Add 400 μl of warm Elution Buffer, mix, and incubate at 65°C for 15 min. Invert every 5 min.

**43**. Centrifuge at >=16,000 × g for 1 min.

**44**. Transfer supernatant to a new 2ml microcentrifuge tube.

**45**. Add another 400 μl preheated Elution Buffer to the sepharose mix, and incubate at 65°C for 15 min. Invert every 5 min.

**46**. Centrifuge at at >=16,000 × g for 1 min.

**47**. Combine second supernatant with the first supernatant.

**48**. Purify and precipitate ChIP DNA exactly as with input DNA.

**49**. Measure concentration. Concentrations are typically close to 1 ng/μl.

**Reagents**

Specialized reagents

- Miracloth (Calbiochem #475855)
- Micrococcal Nuclease (2,000,000 gel units/ml; NEB #M0247S)
- Protease Inhibitor Cocktail (Sigma-Aldrich #9599-1ML)
- rProtein A Sepharose^TM^ Fast Flow (GE Healthcare #17-1279-01)

Stock solutions

- 0.1 *M* potassium phosphate (pH 7.0) = 30.75 ml 1 *M* K_2_HPO_4_ + 19.25 ml 1 *M* KH_2_PO_4_ + 450 ml water
- (1 *M* K_2_HPO_4_ = 8.7 g K_2_HPO_4_ in 50 ml water)
- (1 *M* KH_2_PO_4_ = 6.8 g KH_2_PO_4_ in 50 ml water)
- 1 *M* NaCl = 2.9 g NaCl in 50 ml water
- 1 *M* MgCl_2_ = 10.16 g MgCl_2_ • 6H_2_O in 50 ml water
- 1 *M* CaCl_2_ = 7.35 g CaCl_2_ • 2H_2_O in 50 ml water
- 0.1 *M* PMSF = 0.17 g PMSF in 10 ml ethanol (separate into aliquots and store at -20°C)

Working solutions

M1 Buffer (for two ChIPs)

4 ml 0.1 *M* potassium phosphate (pH 7.0)

4 ml 1 *M* NaCl

4.74 ml hexylene glycol

31.25 μl β-mercaptoethanol

water to 40 ml

M2 Buffer (for two ChIPs)

5 ml 0.1 *M* potassium phosphate (pH 7.0)

5 ml 1 *M* NaCl

500 μl 1 *M* MgCl_2_ 500 μl

5.9 ml hexylene glycol

200 μl Triton X-100

39.05 μl β-mercaptoethanol

water to 50 ml

Elution buffer (for 6 ChIPs)

500 μl 1 *M* NaCl

200 μl 1 *M* Tris-HCl (pH 7.5)

100 μl 0.5 *M* EDTA (pH 8.0)

0.1 g SDS or 500 μl 20% SDS

water to 10 ml

MNB (for 8 ChIPs)

10 ml 50% sucrose

2.5 ml 1 *M* Tris-HCl (pH 7.5)

200 μl 1 *M* MgCl_2_

50 μl 1 *M* CaCl_2_

water to 50 ml

Incubation Buffer (for 8 ChIPs)

1 ml 1 *M* NaCl

400 μl 1 *M* Tris-HCl (pH 7.5)

200 μl 0.5 *M* EDTA (pH 8.0)

water to 20 ml

Wash Buffer A (for 10 ChIPs)

1 ml 1 *M* Tris-HCl (pH 7.5)

400 μl 0.5 *M* EDTA (pH 8.0)

2 ml 1 *M* NaCl

water to 20 ml

Wash Buffer B (for 10 ChIPs)

1 ml 1 *M* Tris-HCl (pH 7.5)

400 μl 0.5 *M* EDTA (pH 8.0)

3 ml 1 *M* NaCl

water to 20 ml
